# Ensuring Global Health Equity in a Post-pandemic Economy: Words Count! Comment on "Ensuring Global Health Equity in a Post-pandemic Economy"

**DOI:** 10.34172/ijhpm.2023.7794

**Published:** 2023-07-24

**Authors:** Mariska Meurs, Myria Koutsoumpa, Valeria Huisman

**Affiliations:** Wemos, Amsterdam, The Netherlands

**Keywords:** Post-pandemic Economy, Health Equity, Green Recovery, Degrowth, Hope-Based Communication

## Abstract

The authors wholeheartedly agree with Labonté: global health equity needs radical changes in economic thinking and policies, including degrowth and reducing consumption in parts of the world. But to mobilize sufficient people for radical change, reducing overconsumption and for degrowth, we may need to stop calling it that. Language is important and using the same frames and words as our opponents do can be counterproductive. Global health advocates need to be strategic about framing, use hope-based communication and develop attractive and convincing narratives. By doing so, hopefully we can bring these messages across to larger groups of people and increase the push for social change.

 In his editorial “Ensuring Global Health Equity in a Post-pandemic Economy,”^1^ Labonté hits the nail on the head: socio-economic inequality and our unsustainable economic growth model are main drivers of health inequity and they require solutions that many consider to be radical. As Labonté describes, the policy tools to implement several of those solutions are already there. Some more and others less elaborated, but still, they are there: a larger role for states in the economy, progressive tax systems — including fair international taxation —, monetary policy reforms and a reform of international financial institutions. Academics have thought them through, activists have been calling for them, and in some cases, politicians are implementing them. What we need, Labonté says, is an activist public health movement to mobilize the political will for implementing these policies. While we wholeheartedly agree, we believe that we need to use different framing, language and words to reach larger groups of people and grow such an activist public health movement that can push for change.

 After hopeful calls for a “green recovery” and “building back better” from many countries and international organisations, as Labonté observes, ambition levels are already declining. In many regions of the world, populist and far-right political groups are gaining votes by electoral promises to continue business as usual. Moreover, civic space is under authoritarian attack in many countries. Meanwhile, in others, ruled by liberal democracy, large groups of voters support political parties whose policy promises do not serve their well-being. Not in the long run and in many cases not even in the short run. Why? And, more importantly, how can we persuade people to support the change we want to see?

 Not by calling for degrowth, for reducing overconsumption or for a radical overhaul — even if we agree with it. The prospect of change, especially radical change, instils feelings of uncertainty and therewith resistance in many people. Even though high-income countries will need to change, a lot, using these terms fosters a feeling that people need to give up something valuable. As Labonté quoted from Walden Bello: *“…there will need to be political and social psychological transformations from societies that have been weaned on overconsumption.” *As activists and part of a social movement, we know that changing the public opinion, is a very complex and multifactorial process.

 To create socio-economic equality and a sustainable economic growth model we need more than an activist public health movement. We need a much more widespread public push for change. To get such a widespread push, we need to weigh our words with care. As global health advocates, we are keenly aware that the framing of a message is often more powerful than its content to convince people and persuade them into behaviour change. However, language, framing and using the words that ‘make people tick’ remains uncharted territory for many of us. While it is exactly the language, framing and words that have the potential to take people to the streets, push for change and possibly change their voting preferences.

 As Anat Shenker-Osorio says: *“A great message doesn’t say what’s already popular; a great message makes popular what needs to be said.”*^2^

 Therefore, we are making a case for using convincing framing and hope-based communication. Research shows that framing is essential to convince people of your message. Cognitive scientist George Lakoff explains it like this: *“facts matter enormously, but to be meaningful they must be framed in terms of their moral importance. […] If the facts don’t fit the frames in your brain, the frames in your brain will stay and the facts are ignored or challenged or belittled.”*^3^ So, people’s brains take shortcuts to interpret what you are saying, and those shortcuts are based on the ideas they already have.

 Often, advocates try to counter a narrative by using the same words as their opponents. Think of the ‘Brexit’ versus ‘No Brexit’ campaign. This is counterproductive. When using words that strongly link to the opposite frame, you activate that frame, undermining your own views. It is important to use your own frames, choose your own words, not those of the ones holding a different view. According to Lakoff, effective reframing is more than presenting the facts in an effective way. It is about ingraining certain ideas, developed over time, consistently and precisely enough to create an accurate frame for our understanding.^3^

 Hope-based communication builds on that idea. It emphasizes the importance of creating strong, positive narratives (frames) based on our shared values. At a high level, our values are quite similar; we all want to be as healthy as possible; we want the best for our children; and we desire to be loved and treated with respect. As Bonanno et al say: *“[These] shared values are widely held beliefs among the population of interest.” *and* “[serve] to build a connection between the speaker and the audience, creating a willingness to listen to further information.”*^4^ A telling example to illustrate this point is given by Anat Shenker-Osorio: *“Marriage equality won out precisely because LGBT people made the debate about values of commitment and family. When they stopped talking ‘rights’ and started talking ‘love,’ the tide turned.”*^2^

 Next, we must paint a clear and appealing picture of what our ideal world looks like. If people recognize themselves in that view, it becomes easier for them to follow ideas and call for or adopt policies that will help realize this world. Thomas Coombes, communication strategist and hope-based communication champion, explains that hope-based communication does not ignore the problems, but instead it puts them into the context of how things should be.^5^ So, rather than reacting to our opponents’ ideas — merely focusing on what we are against —, we must show that it is possible to make the changes, offering a hopeful perspective that is activating.^6^ For example, research in the environmental field shows that people are more likely to change their intentions when they receive a positive framing of an issue, whereas fear can leave them overwhelmed and not action oriented.^7-9^

 We recognize that there is no silver bullet when it comes to effecting social change. However, we do think, and evidence supports, that creating a positive perspective is more activating than focusing only on the problems.^10^ And thus, worth exploring in our quest to realize global health equity.

## Redirecting the Growth Narrative

 Let’s look at the framing around economic growth and degrowth. People often relate growth with something positive, like improvements in health and well-being. And when we think about economic growth, the shortcut in our brains usually leads us to the most common, most used indicator for it: gross domestic product (GDP). We quickly link GDP with positive outcomes, not leaving room to reflect that focusing on GDP growth without taking measures to equitably distribute wealth and invest in the social sectors, will not be beneficial for all of us. Or that GDP also grows as a result of activities that are downright harmful, to the environment, to health, to the well-being of many. The United States, for example, stands out as a country with one of the highest GDP growth rates and GDP per capita in the world. Nevertheless, among other GDP-high countries, it also has the highest economic inequality and poverty, and lack of universal access to healthcare, heavily influencing the life expectancy and well-being of the population.

 In fact, economic equality correlates far more closely with happiness, longevity and well-being of the population than GDP. According to the World Health Organization (WHO),^11^ evidence shows that even a modest redistribution of wealth has considerably greater impact on poverty reduction than economic growth alone. And according to Wilkinson and Pickett, it is not so much the growth of an economy that matters, but rather how wealth is distributed within it.^12^ Economic growth does not, in itself, improve well-being. Tax revenues may indeed increase with GDP growth, but what matters is whether and how governments invest those revenues in good quality and universally accessible health and education, infrastructure and other public services.

 Unfortunately, the Sustainable Development Goals (SDGs) have legitimized the use of GDP as the most appropriate economic indicator. It has become and continues to be part of our vocabulary as civil society and in the global discourse, as we often refer to the SDGs as ‘the world we want.’ But if the world we want is fair and just, with well-being for everyone, we need to measure different things.

 Alternatives exist and have for many years. They can be very useful for building a new narrative of what our ideal world looks like. The Genuine Progress Indicator (GPI) is one. It starts with a measurement of GDP but then considers positive externalities like household and volunteer work, and subtracts negative externalities, such as pollution, resource depletion and crime; and it adjusts for inequality.^13^ So, it basically tries to net the positive and negative outcomes of economic growth to evaluate whether or not it has benefited society. Another alternative is Bhutan’s Gross National Happiness indicator (GNH). The central concept of GNH is that sustainable development should give equal importance to non-economic aspects of well-being, like sustainable and equitable socio-economic development. We need to move away from the eternal chase of GDP and growth as we know it.

 If governments and the global community would shift their policies and approach towards maximising the GPI or GNH or any other sustainable indicator instead of GDP, then they would adopt policies that improve social well-being and allow for a fairer distribution of wealth, and health and well-being, across the world.

 How do we get them to do that, when economic and GDP growth continues to dominate the headlines of major news channels and to drive decision-making? Well-framed information is only one piece of the puzzle of change, which is a wide and complex territory that social movements, including the global health community, are still trying to fully grasp. Knowledge is important, but change is a dynamic, iterative process that also differs across contexts and time.

 To garner the widespread public support that is needed, we must create strong and convincing framing. Let’s be deliberate and creative with words. Terms like “no Brexit,” or even “degrowth,” do not convey a vision of the world you want to create, instead it activates and strengthens the opposite view. Do not assume people think from the same starting point as you do. Keep emphasizing what you want the world to look like and why – linking to our shared values. Once people share your frame, your ideas for change will stick much better. We must find the right words and the right frames to help make that happen.

 Rather than calling for ‘degrowth,’ let’s call for growing a care economy, as suggested by one of the interviewees of Labonté in his article. Instead of emphasizing the need to reduce consumption, we can focus on the need for increased consumption of what is essential for well-being, such as clean air and universal health coverage. If we can paint a picture – in our own words – of a world in which all can flourish, then hopefully we can activate people at the grassroots level to bring change from below and sufficient people to vote into office those political leaders that will raise and maintain ambition levels for a green, caring and inclusive economy.

## Ethical issues

 Not applicable.

## Competing interests

 Authors declare that they have no competing interests.

## Disclaimers

 The views expressed in this commentary are those of the authors and do not constitute an official position of Wemos.

## Funding

 All authors are employees of Wemos, a non-profit organization. Wemos’ sources of funding can be found at https://www.wemos.org/.
